# Sustained hyperosmolarity increses TGF-ß1 and Egr-1 expression in the rat renal medulla

**DOI:** 10.1186/s12882-017-0626-2

**Published:** 2017-07-03

**Authors:** Miklós M. Mózes, Petra Szoleczky, László Rosivall, Gábor Kökény

**Affiliations:** 1 Institute of Pathophysiology, Semmelweis University, Nagyvárad tér 4, Budapest, H-1089 Hungary; 2 Hungarian Academy of Sciences and Semmelweis University Research Group for Pediatrics and Nephrology, Budapest, Hungary

**Keywords:** Osmotic stress, Urea, Sodium chloride, Fibrosis, TGF-ß, Egr-1

## Abstract

**Background:**

Although TGF-ß and the transcription factor Egr-1 play an important role in both kidney fibrosis and in response to acute changes of renal medullary osmolarity, their role under sustained hypo- or hyperosmolar conditions has not been elucidated. We investigated the effects of chronic hypertonicity and hypotonicity on the renal medullary TGF-ß and Egr-1 expression.

**Methods:**

Male adult Sprague Dawley rats (*n* = 6/group) were treated with 15 mg/day furosemide, or the rats were water restricted to 15 ml/200 g body weight per day. Control rats had free access to water and rodent chow. Kidneys were harvested after 5 days of treament.

In cultured inner medullary collecting duct (IMCD) cells, osmolarity was increased from 330 mOsm to 900 mOsm over 6 days. Analyses were performed at 330, 600 and 900 mOsm.

**Results:**

Urine osmolarity has not changed due to furosemide treatment but increased 2-fold after water restriction (*p* < 0.05). Gene expression of TGF-ß and Egr-1 increased by 1.9-fold and 7-fold in the hypertonic medulla, respectively (*p* < 0.05), accompanied by 6-fold and 2-fold increased c-Fos and TIMP-1 expression, respectively (*p* < 0.05) and positive immunostaining for TGF-ß and Egr-1 (*p* < 0.05).

Similarly, hyperosmolarity led to overexpression of TGF-ß and Egr-1 mRNA in IMCD cells (2.5-fold and 3.5-fold increase from 330 to 900 mOsm, respectively (*p* < 0.05)) accompanied by significant c-Fos and c-Jun overexpressions (*p* < 0.01), and increased Col3a1 and Col4a1 mRNA expression.

**Conclusion:**

We conclude that both TGF-ß and Egr-1 are upregulated by sustained hyperosmolarity in the rat renal medulla, and it favors the expression of extracellular matrix components.

**Electronic supplementary material:**

The online version of this article (doi:10.1186/s12882-017-0626-2) contains supplementary material, which is available to authorized users.

## Background

Chronic kidney disease counts for a significant number of deaths worldwide, and its prevalence is estimated to be 8–16% [1]. The pathomechanisms of glomerulosclerosis and tubulointerstitial fibrosis show many similarities irrespective of the etiology (eg. diabetes mellitus, hypertension, etc.) [2].

Tranforming growth factor-ß (TGF-ß) has a major impact on fibrotic diseases, including myocardial, pulmonary and kidney fibrosis [3–6]. TGF-ß is present in the inner medulla of humans and rats, inhibiting sodium transport by the inner medullary collecting duct (IMCD) cells [7–10]. High water intake ameliorates tubulointerstitial injury by reducing TGF-ß mRNA expression in the medullary interstitium [11], whereas high salt diet in Sprague Dawley rats increased the renal expression and urinary excretion of TGF-ß, without altering plasma TGF-ß levels [12]. In renal fibroblasts, acute increase of hyperosmolarity resulted in TGF-ß activation in vitro [13]. TGF-ß can induce the transcription of several genes, including the transcription factors Egr-1 (early growth response factor-1) and the activator protein-1 (AP-1), which has been associated with upregulation of extracellular matrix production [14–16]. AP-1 can induce extracellular matrix accumulation also through the activation of the tissue inhibitor of metalloprotease-1 (TIMP-1) expression [17]. Short term osmotic stress has been reported to upregulate Egr-1 [18–20] in vitro but little is known about its role in the renal medulla. The transcription factor AP-1 is a heterodimeric molecule, composed of Jun and Fos nuclear oncoprotein families, being c-Jun and c-Fos the two major components of AP-1 [21].

As sustained hypertonicity is present in the renal medulla, we aimed to investigate how altered medullary osmolarity (by increasing or decreaseing solute) might affect TGF-ß, Egr-1 and AP-1 expressions. We examined hyperosmolar medulla by 60% water restriction, which resulted in avid distal tubule reabsorption thus increased medullary tonicity. Hypoosmolar medulla was achieved by furosemide treatment to increase urine flow rate and deplete medullary solute [22]. We have also confirmed our in vivo results on murine inner medullary collecting duct (IMCD) cells cultured at 330, 600 and 900 mOsm.

## Methods

### Animals

Eighteen male Sprague-Dawley rats weighing 250 to 300 g (Crl:SD, Charles River, Sulzfeld, Germany) were housed individually in polycarbonate cages (Type 1290 L, Tecniplast, Buguggiate, Italy) in a barrier room at a constant temperature of 22 ± 2 °C with 12 h light/dark cycles. The number of animals was calculated by the Ethical Committee to provide the minimum sample size needed for relevant results. Rats were randomly distributed into three experimental groups. Standard rodent chow and water access varied according to the experimental groups, as follows:

Control rats (*n* = 6) received 15 g chow and 18.5 ml of water per 100 g of body weight; Water restriction group (*n* = 6) received 15 g chow and 7.5 ml of water per 100 g of body weight; rats in the Furosemide group (*n* = 6) had access to chow and water ad libitum, but were treated with furosemide (at a dose of 5 mg/kg) twice daily for 5 days [22], and also had free access to 0.8% NaCl/0.1% KCl drinking water to replace salt losses.

Twenty-four hour collected urine samples were obtained using metabolic cages (Tecniplast). Baseline collected urine and blood samples from retroorbital plexus were collected on the first day at 8 AM, and repeatedly on day 5 at 8 AM, when kidneys were harvested after euthanasia with carbon dioxide in a closed container. The medulla and cortex of the right kidney were separated and snap frozen, while the left kidney was cut in half and fixed in 4% buffered formalin solution overnight, then embedded in paraffin.

### Urine chemistries

Urine samples were collected for 24 h in metabolic cages at day 0 and day 7 of the study, and urine volume (V) was determined. Urine creatinine concentrations were measured photometrically using commercial kits (Diagnosztikum Zrt, Budapest, Hungary). Urine protein concentration was measured (BCA Protein Assay, Pierce Thermo Scientific, Rockford, USA), and urine protein/creatinine ratios were calculated. Urine osmolarity (Osm_urine_) was measured by standard laboratory osmometer in the Central Laboratory of Semmelweis University and the amount of daily excreted osmotic material (mOsm/24 h) was calculated as: Osm_urine_ (mOsm/l) x V (liter/24 h).

### Renal immunohistochemistry

Immunohistochemistry was performed on formalin fixed, paraffin embedded sections, using the avidin-biotin method, as previously described [23]. Briefly, after antigen retrieval in citric buffer, slides were incubated overnight at 4 °C with rabbit polyclonal anti-TGF-β1 (1:100; Santa Cruz Biotechnology, Santa Cruz, CA, USA) or rabbit polyclonal EGR-1 (1:400, Cell Signaling Technologies) antibodies. Secondary antibodies (SuperSensitive Link) were purchased from BioGenex (San Ramon, CA, USA). The slides were developed with Liquid Permanent Red (Dako). Negative controls for all immunostainings were performed by omitting the primary antibodies. Nuclei were counterstained with hematoxylin solution, then slides were mounted using Aquatex (Merck) and analyzed under light microscope.

Immunohistochemical staining of the inner and outer medulla was examined at a magnification of ×400 counting the number of positive cells per field in each section, as described previously [23]. All samples were evaluated in a blind manner.

### Cell culture of inner medullary collecting duct (IMCD) cells

Murine IMCD cells (mIMCD-3) were purchased from American Type Culture Collection (ATCC Catalogue# CRL-2123, Manassas, VA, USA) and maintained in Dulbecco’s modified Eagle’s medium (DMEM, Sigma-Aldrich, Budapest, Hungary) containing 1 g/l glucose supplemented with 10% fetal bovine serum (Gibco, Life Technologies, USA), 4 mM glutamine and 100 IU/ml penicillin and 100 μg/ml streptomycin (Gibco) and cultured for at 37 °C in 5% CO_2_ atmosphere.

IMCD cells were plated on 6 cm dishes (500,000 cell/dish) and cultured in DMEM/F12 medium (Sigma-Aldrich) containing 10% FBS and antibiotics. For the MTT assay, cells were plated on 96-well plates (25,000 cells/well). After confluency, the medium was switched to serum-free DMEM/F12 medium supplemented with 3,3′,5-Triiodo-L-thyronine (T3, Sigma-Aldrich), insulin-transferrin-selenium (ITS-G, Gibco), Bovine Serum Albumin (Sigma-Aldrich) and hydrocortisone (Sigma-Aldrich). In order to mimic the hyperosmolar conditions of the renal medulla, the medium osmolarity was gradually increased by adding NaCl and urea, from 330 mOsm reaching 900 mOsm on day 7. At specific osmolarities (330, 600, 900 mOsm), cells were harvested for MTT assay (on 96-well plates, *n* = 8 replicates/group), RNA and protein isolation (on 6 cm dishes, *n* = 4 replicates/group).

### MTT viability assay

To estimate the number of viable cells 3-(4,5-dimethyl-2-thiazolyl)-2,5-diphenyl-2H-tetrazolium bromide (MTT) was used. First the cells were dissociated with 0.05 mM EDTA (at a final concentration of 2.5 μM) at 37 °C for 15 min at 5% CO2 atmosphere to allow complete dye uptake, then FBS containing 3-(4,5-dimethyl-2-thiazolyl)-2,5-diphenyl-2H-tetrazolium bromide (MTT, Calbiochem, EMD BioSciences, San Diego, CA, USA) was added in 1/10 volume to reach final concentration of 0.5 mg/mL, and the cells were incubated for 3 h at 37 °C at 5% CO2 atmosphere [24]. Cells were washed with PBS, dried overnight at room temperature and the formazan dye was dissolved in isopropanol. The amount of converted formazan dye was measured at 570 nm with background measurement at 690 nm on a Powerwave reader (Biotek). The calibration curve created with serial dilutions of IMCD cells was used to calculate the viable cell count with the Gen5 data reduction software (Biotek, Winooski, VT, USA).

### Immunocytochemistry

IMCD cells (5000 cells/well) were seeded on BD multichamber slides and underwent the gradual increase in osmolarity as described above. At 330, 600 and 900 mOsm, cells were washed with ice-cold PBS and fixed with 4% paraformaldehyde for 30 min at room temperature. After washing with PBS, cells were blocked using 1× Powerblock (Biogenex, San Ramon, CA, USA) for 10 min and incubated with rabbit polyclonal TGF-ß1 antibody (1:50 in PBS, Santa Cruz) overnight at 4C. The slide was carefully washed twice with PBS and incubated with secondary antibody (Rabbit Link, Biogenex) for 30 min, washed and developed with Fast Red (Dako). Nuclei were counterstained with hematoxylin solution, then slides were mounted using Aquatex (Merck) and analyzed under light microscope.

### Immunoblot

Cells were harvested in RIPA lysis buffer containing complete protease inhibitor cocktail (Roche). Protein concentration was determined by the BCA Assay. Samples were mixed with 2× Laemmli buffer and boiled. Equal amounts of protein (20 μg) were separated on 12% SDS-polyacrylamide gel, transferred to nitrocellulose membranes and blocked with 5% skim milk in Tris-buffered saline (TBS), containing 0.1% Tween-20. Membranes were incubated overnight at 4 °C with rabbit polyclonal TGF-ß1 (1:250, Santa Cruz Biotechnology, Santa Cruz, USA) or mouse monoclonal tubulin antibody (1:10,000, Sigma-Aldrich), washed and incubated with peroxidase-conjugated secondary antibody (anti-mouse IgG or anti-goat IgG, 1:5000, Cell Signaling,). Blots were visualized by ECL detection kit (Pierce Thermo).

### Quantitative RT-PCR

IMCD cells for gene expression analysis were harvested with using 1 ml of Trizol reagent per well (Life Technologies, USA) for phenol/chloroform extraction of total RNA according to the manufacturer’s protocol. For renal gene expression analysis, 100 mg of whole kidneys were homogenized and total RNA was isolated according to the manufacturer’s protocol (SV Total RNA kit, Promega, Madison, WI, USA). From both IMCD and kidney samples, 2 μg RNA was reverse transcribed (High Capacity cDNA Reverse Transcription kit, Applied Biosystems, Forster City, CA, USA) using random primers. PCR reactions were performed on a BioRad CFX thermal cycler (BioRad, Hercules, CA, USA) using the Power SYBR Green PCR Master Mix (Applied Biosystems). Specificity and efficiency of the PCR reaction was confirmed with melting curve and standard curve analysis, respectively. Duplicate samples were normalized to glyceraldehyde-3-phosphate dehydrogenase *(Gapdh)* expression. Mean values are expressed with the formula 2^-ΔΔCt^.

Mouse primer sequences were as follows: *mCol3a1 forward* 5-TGGAAAAGATGGAACAAGTGG-3; *mCol3a1 reverse* 5-CCAGACTTTTCACCTCCAAC-3; *mCol4a1 forward* 5-CCTGCTAATATAGGGTTCGAG-3; *mCol4a1 reverse* 5-CCAGGCTTAAAGGGAAATCC-3; *mEgr1 forward* 5-TTCAATCCTCAAGGGGAGCC-3; *mEgr1 reverse* 5-TAACTCGTCTCCACCATCGC-3; *mTgfb1 forward* 5-CACCATCCATGACATGAACC-3; *mTgfb1 reverse* 5-TCATGTTGGACAACTGCTCC-3.

Rat primer sequences were as follows: *rCol3a1 forward:* 5-GAAGTCTCTGAAGCTGATGGG-3*; rCol3a1 reverse:* 5-GGCCTTGGGTGTTTGATATTC-3*; rTgfb1* forward 5-ACCATCCATGACATGAACC-3; *rTgfb1* reverse 5-TCATGTTGGACAACTGCTCC-3; *rTimp1* forward: 5-TTTCTGCAACTCGGACCTG-3; *rTimp1* reverse: 5-ACAGCGTCGAATCCTTTGAG-3; *rGapdh* forward: 5-CAATGACCCCTTCATTGACC-3; *rGapdh* reverse: 5-CGCCAGTAGACTCCACAACA-3.

Primer sequences common for mouse and rat genes were as follows: *cFos forward* 5-TTTCAACGCCGACTACGAGG-3; *cFos reverse* 5-GCGCAAAAGTCCTGTGTGTT-3; *cJun forward* 5-GCACATCACCACTACACCGA-3; *cJun reverse* 5-GGGAAGCGTGTTCTGGCTAT-3.

### Statistics

All data are presented as mean ± SD. One-way analysis of variance (ANOVA) followed by Tukey’s post-hoc test, Kruskal-Wallis test followed by Dunn’s post-hoc test or Mann-Whitney test were applied as appropriate with the significance level set to *p* < 0.05 (SPSS 13 for Windows, SPSS Inc., USA).

## Results

### Water restriction induces TGF-ß overexpression in the rat medulla

**Fig. 1:**
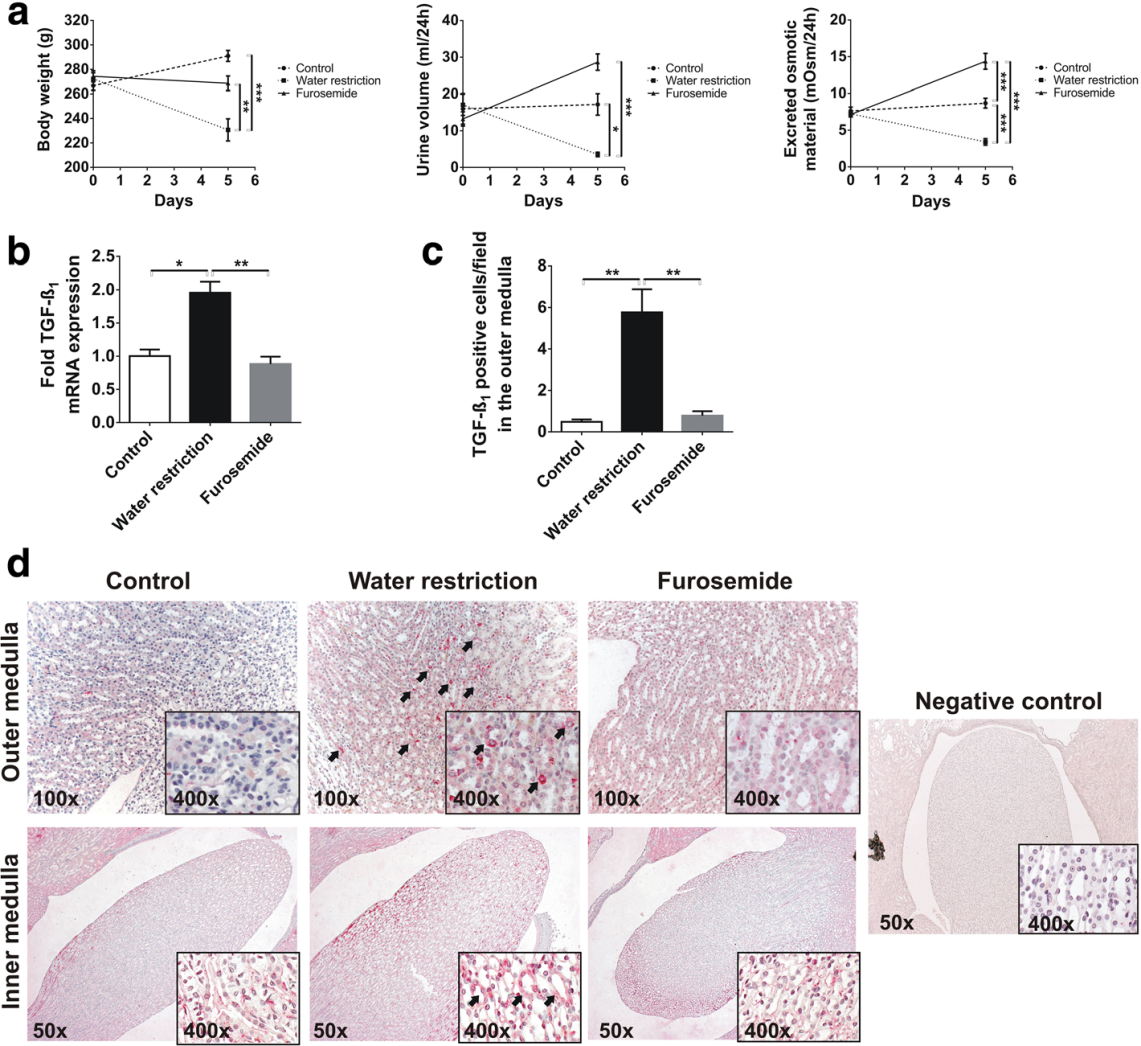
Effect of increased or decreased medullary solute concentration in rats. **a** Compared to the initial body weights and to controls, both water restricted and furosemide treated rats had significantly lower body weights at harvest. The urine volume dropped by 50% due to water restriction, but increased almost 2-fold in furosemide treated rats, accompanied by significantly decreased or increased excretion of solutes, respectively. **b** Water restriction increased mRNA expression of TGF-ß by 2-fold in the medulla, accompanied by marked TGF-ß immunostaining **c**, **d**, as compared to control and furosemide treated rats. **d** The cytoplasm of outer medullary tubular epithelial cells were negative in controls and only weakly stained in furosemide treated rats, but no interstitial staining was observed. However, water restriction increased the cytoplasmic TGF-ß staining intensity in several tubular cells in the inner stripe of the outer medulla (*red cytoplasm*, see *arrows*). In contrast, TGF-ß immunostaining showed mostly interstitial staining in the inner medulla of controls and furosemide treated rats, but also tubular cytoplasmic staining in water restricted rats (see *insets*, arrows pointing on positive cells). Primary antibody was omitted as negative control for immunostaining. Data are presented as mean ± SEM, *n* = 6/group. * *p* < 0.05, ** *p* < 0.01, *** *p* < 0.001 (one-way ANOVA with Tukey’s post-hoc test or Kruskal-Wallis test with Dunn’s post-hoc test)

During the 5 day follow-up, the body weight of both controls and furosemide treated (hypoosmolar) rats remained unchanged, but water restricted (hyperosmolar) rats showed a moderate but significant weight loss (Fig. 1a). The effectiveness of water restriction or furosemide treatment was evaluated by daily urine volume and urine osmolarity measurements at the beginning (day 0) and at the end of the study (day 5). The volume of twenty-four hour collected urine remained unchanged in controls, whereas decreased dramatically in hyperosmolar rats but increased in hypoosmolar (furosemide treated) rats (Fig. 1a). The daily amount of excreted osmotic material showed similar changes.

TGF-ß mRNA expression in the medulla of water restriucted rats increased by 2-folds as compared to control and furosemide treated rats (Fig. 1b), and similar trend was observed by evaluation of TGF-ß immunohistochemistry (Fig. 1c). We postulate the relatively higher fold changes in TGF-ß protein expression as an activation of latent TGF-ß, in addition to de novo TGF-ß synthesis. The inner stripe of the outer medulla and fornix showed TGF-ß positive cells only in water restricted rats (Fig. 1d, outer medulla, see arrows), but no outer medullary staining in controls and furosemide treated rats. In contrast, TGF-ß immunohistochemistry did not show marked cytoplasmic immunoreactivity of the tubular epithelial cells in the papilla and inner medulla of controls and furosemide treated rats, but only interstitial staining. Water restricted rats, however, depicted more intensive cytoplasmic TGF-ß immunostaining in the inner medulla (Fig. 1d, inner medulla).

### Chronic increase in medullary osmotic concentration induces overexpression of profibrotic transcription factors

**Fig. 2:**
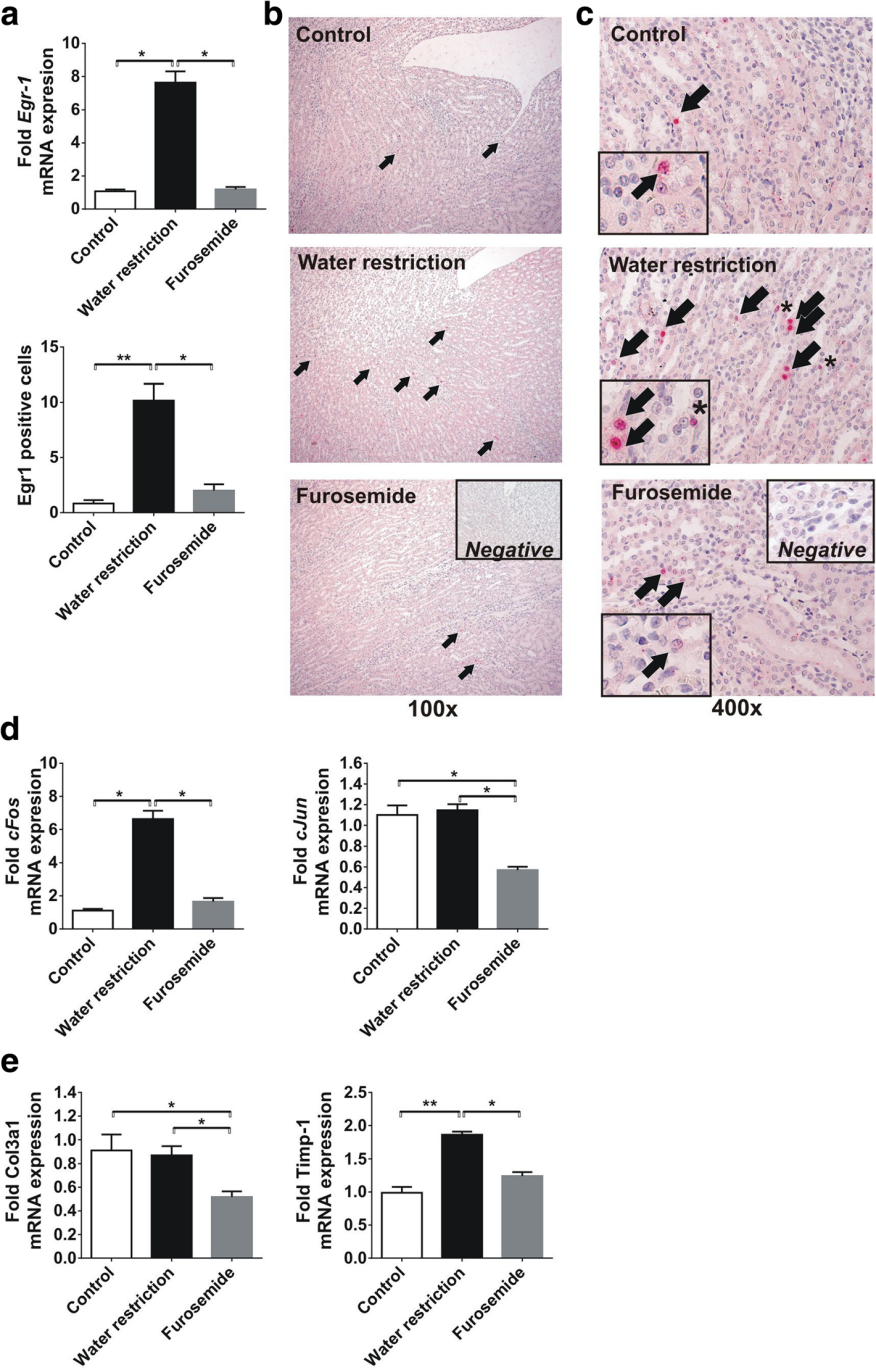
Expression of profibrotic transcription factors due to chronic increased or decreased medullary solute concentration in rats. **a** Compared to control rats, water restriction increased the medullary expression of Egr-1 mRNA and protein, while furosemide treated rats had similar expression levels to controls. **b** The Egr-1 overexpression was confirmed by immunohistochemistry, depicting a significant amount of Egr-1 positive tubular cell nuclei in water restricted rats (*arrows*), as compared to both control and furosemide treated rats. **c** Interestingly, apart of the tubular staining (*arrows*), we also found Egr-1 positive interstitial cells only in the medulla of water restricted rats (*asterisk*). **d** Water restriction significantly increased the mRNA expression of the AP-1 component cFos. The expression of the AP-1 component cJun did not change due to increased medullary solute concentration, but decreased by 50% after furosemide treatment. **e** Increased medullary osmolarity had no effect on the mRNA expression of collagen-III, but it was reduced by furosemide treatment. As compared to controls, the medullary TIMP-1 expression was almost 2-fold elevated after chronic water restriction, and furosemide did not alter TIMP-1. Data are presented as mean ± SEM, *n* = 6/group. * *p* < 0.05, ** *p* < 0.01, *** *p* < 0.001 (Kruskal-Wallis test with Dunn’s post-hoc test)

Renal mRNA expression of the transcription factor Egr-1 was more than seven-fold upregulated in rats on water restriction, but remained at control levels in furosemide treated rats (Fig. 2a). Additionally, Egr-1 immunohistochemistry revealed also a significant amount of positive renal medullary cells in water restricted rats, compared to barely no staining in controls or furosemide treated animals (Fig. 2a–c). Interestingly, Egr-1 staining in control and hypoosmolar kidneys was restricted to tubular cells (Fig. 2c), whereas in hyperosmolar kidneys we observed both tubular (arrows) and interstitial (asterisk) Egr-1 expression.

Chronic water restriction led to more than six-fold overexpression of the AP-1 component c-Fos. Interestingly, water restriction did not alter c-Jun mRNA expression, but furosemide reduced c-Jun expression by 50% as compared to controls while had no effect on cFos expression (Fig. 2d).

Similarly to cJun, type III collagen mRNA expression was not altered by water restriction but was repressed by furosemide treatment (Fig. 2e). However, the chronic increase in medullary osmotic concentration in water restricted rats induced TIMP-1 overexpression by 2-fold, while the low osmotic concentration due to furosemide treatment did not influence TIMP-1 (Fig. 2e).

### Cell viability of IMCD cells decreases with hyperosmolarity

**Fig. 3:**
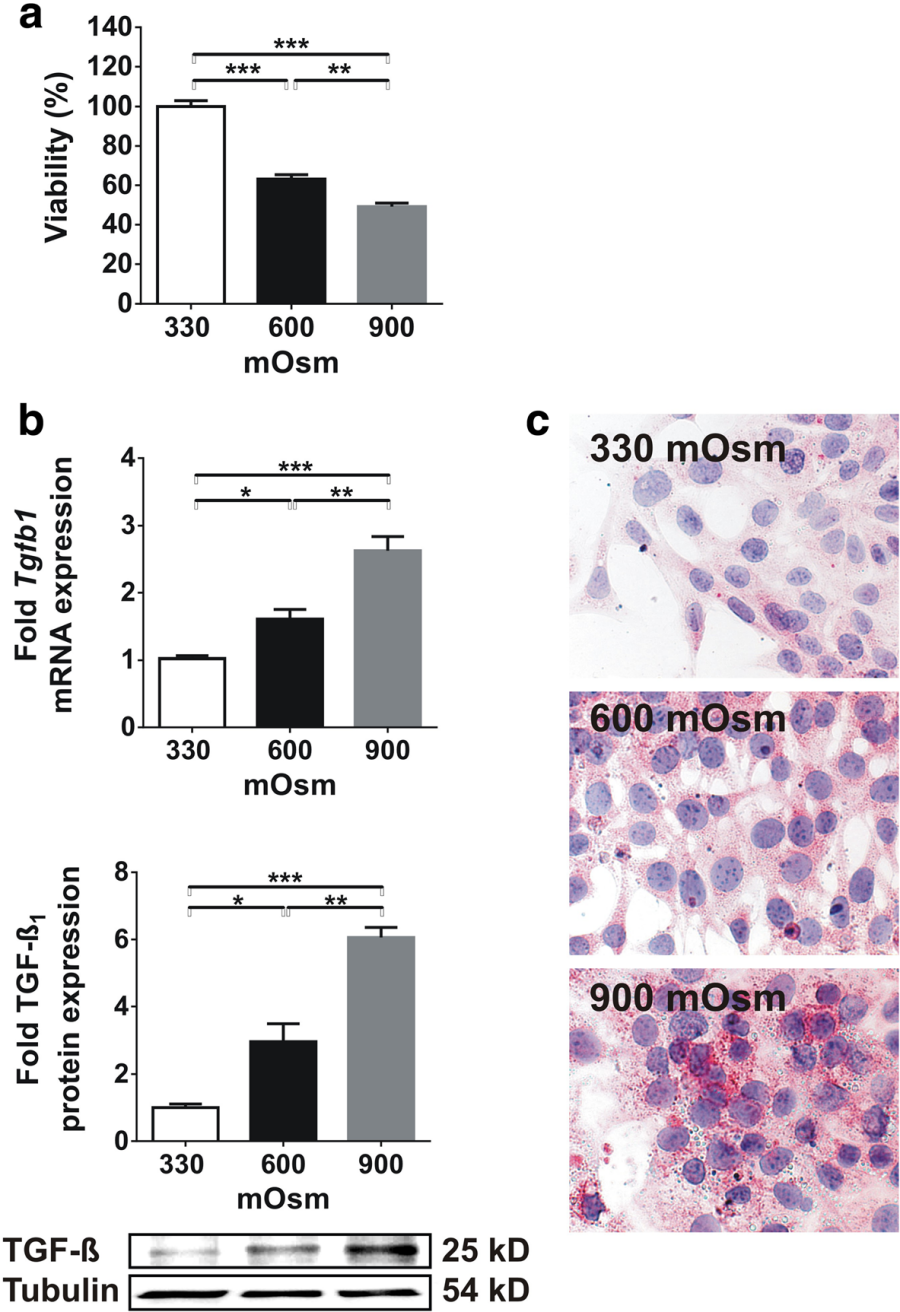
Effect of chronic increase in medium osmolarity on viability and TGF-ß expression of IMCD cells. **a** Chronic hyperosmolarity significantly reduced the viability of IMCD cells (*n* = 8/group). **b** The expression of TGF-ß, however, increased dramatically both at mRNA and protein level (*n* = 4/group). **c** Accordingly, immunocytochemistry depicted significant TGF-ß staining at 600 mOsm and even stronger staining at 900 mOsm (*n* = 3/group). Data are presented as mean ± SEM. * *p* < 0.05, ** *p* < 0.01, *** *p* < 0.001 (one-way ANOVA with Tukey’s post-hoc test)

Based on the in vivo results, we also wished to study the effect of hyperosmolarity on IMCD cells. In vitro, the progressive increase of osmolarity resulted in decreased viability of IMCD cells as shown by MTT assay. Compared to controls (at 330 mOsm), reaching 600 mOsm in the medium led to 40% fall in viabilty, and a further 10% decrease was observed when we increased the osmolarity to 900 mOsm (Fig. 3a).

### Hyperosmolarity induces TGF-ß overexpression in IMCD cells

In parallel with the decreased viability, we observed marked increase in TGF-ß mRNA and protein expressions due to hyperosmolarity. TGF-ß mRNA expression increased from 330 mOsm to 600 mOsm and from 600 to 900 mOsm by 1.5 fold and further 1.6 fold, respectively (Fig. 1b). This was accompanied by a similar increase in TGF-ß protein expression by 3-fold and 2.4-fold, respectively (Fig. 3b). This observation was confirmed by immunocytochemistry as well (Fig. 3c).

### Increased osmolarity induces the expression of profibrotic genes in IMCD cells

**Fig. 4:**
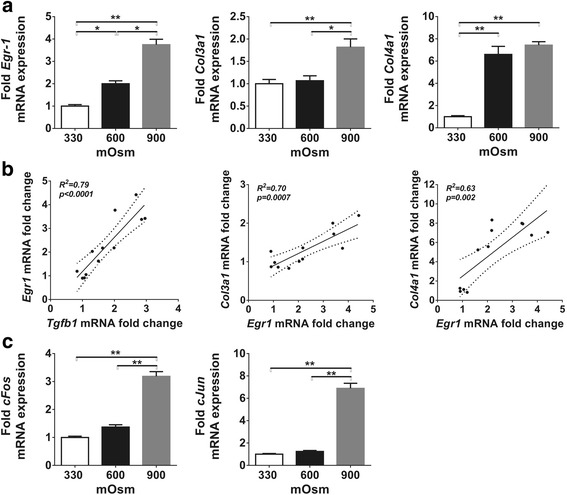
Effect of chronic hyperosmolarity on the expression of profibrotic genes and transcription factors in IMCD cells. **a** The progressive increase of medium osmolarity induced overexpression of the Egr-1 mRNA. Mild type III collagen upregulation was induced only by 900 mOsm, dramatic (6-fold) type IV collagen overexpression was observed at medium osmolarity of 600 mOsm. **b** Egr-1 and TGF-ß mRNA expressions showed the strongest correlation. Both type III and type IV collagen expressions correlated well to the expression of the profibrotic Egr-1. **c** Components of the transcription factor AP-1, cFos and cJun, were strongly overexpressed only at high osmolar concentration of 900 mOsm. Data are presented as mean ± SEM, *n* = 4/group. * *p* < 0.05, ** *p* < 0.01 (Kruskal-Wallis test with Dunn’s post-hoc test)

As TGF-ß is one of the key growth factors that participate in the pathomechanism of fibrosis, we investigated whether its increased expression due to hyperosmolarity could contribute to fibrotic gene expression in vitro. Surprisingly, the progressive increase in osmotic concentration led to a gradual 2-fold increase in early growth response factor-1 (Egr-1) expression, accompanied by mild and late overexpression of collagen-3 but marked and early overexpression of collagen-4 (Fig. 4a). The mRNA expression of Egr-1 correlated well to TGF-ß (*p* < 0.0001, Fig. 4b) but Egr-1 also correlated significantly to both type III and type IV collagen expressions (Fig. 4b), which indicates that hyperosmolarity might induce extracellular matrix overproduction via activation of Egr-1.

The members of AP-1 transcription factor, c-Fos and c-Jun were significantly upregulated at sustained hyperosmolarity reaching 900 mOsm, but remained unchanged at 600 mOsm (Fig. 4c).

### TGF-ß induces Egr-1 and collagen-IV production in IMCD cells

**Fig. 5:**
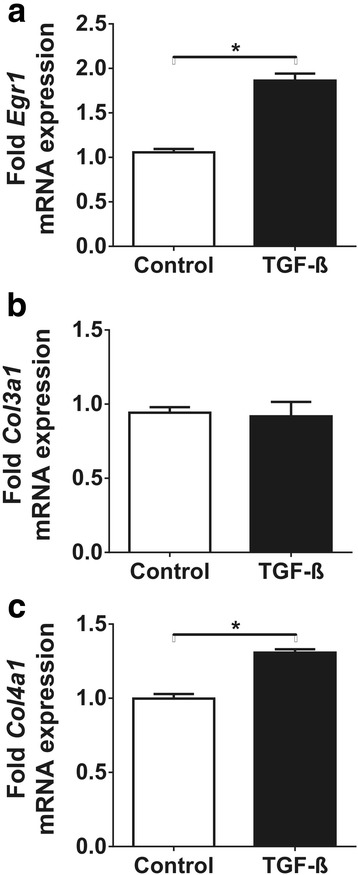
Effect of TGF-ß administration on IMCD cells under normal osmotic concentration. TGF-ß administration for 48 h significantly upregulated Egr-1 mRNA expression by 2-fold (**a**) but had no effect on type III collagen expression (**b**). As compared to the effects of increased osmotic concentration, TGF-ß administration induced only a mild type IV collagen overexpression (**c**). Data are presented as mean ± SEM, *n* = 3/group. * *p* < 0.05 (Mann-Whitney test)

In order to elucidate whether the overexpression of profibrotic genes in IMCD cells under hyperosmolar environment are mainly a result of increased TGF-ß, we investigated the effect of direct TGF-ß administration on IMCD cells in normal osmolarity. TGF-ß treatment of IMCD cells resulted in 2-fold increased Egr-1 mRNA expression (Fig. 5a) accompanied by only mild, 30% increase in type IV collagen expression (Fig. 5c). Surprisingly, type III collagen expression of TGF-ß treated cells was similar to non-treated controls (Fig. 5b). This experiment shows that even a mild hyperosmolarity of 600 mOsm exerts more potent effect on IMCD gene expression as compared to the potent profibrotic TGF-ß.

## Discussion

The present study shows that sustained hypertonicity induces the expression of TGF-ß and Egr-1 in the renal medulla, both in vivo and in vitro. The renal medulla of water restricted rats showed Egr-1 and AP-1 overexpression, increased amount of Egr-1 expressing tubules as well as interstitial cells, which was abrogated by furosemide treatment. Hypertonicity induced Egr-1 in IMCD cells correlated with type III and type IV collagen expressions but TGF-ß treatment resulted in only mild type IV collagen overexpression. Our study shows that sustained hypertonicity facilitates the production of extracellular matrix components through Egr-1 overexpression and AP-1 activation.

It is well known that TGF-ß has a major impact on kidney fibrosis [5, 6]. However, less is known about its role in tubular function. TGF-ß is present in the inner medulla, inhibiting sodium transport by the inner medullary collecting duct (IMCD) cells [9]. TGF-ß can induce the trascription of several genes, including the transcription factor Egr-1 (early growth response factor-1) which has been associated with upregulation of extracellular matrix production [25, 26]. Egr-1 overexpression of IMCD cells in vitro induced by acute changes in osmolarity implicates an important role of Egr-1 in cellular responses against osmolar stress. Short term (10 or 30 min) hyperosmotic urea treatment has been reported to upregulate Egr-1 in IMCD cells [18–20]. On the other hand, acute hypotonic stress of IMCD cells for 6 h also increased Egr-1 expression [27].

In the renal medulla, a fluctuating but sustained hypertonicity is present [28, 29], therefore we postulated that osmotic concentration might regulate TGF-ß and Egr-1 expression of renal medullary cells in vivo as well. The sustained water restriction of rats induced overexpression of both TGF-ß and Egr-1 in the renal medulla, accompanied by increased TIMP-1 and c-Fos expression. These changes were completely abolished when the osmotic gradient was washed out by furosemide treatment. Water restriction increased the cytoplasmic TGF-ß staining intensity in several tubular cells in both the inner stripe of the outer medulla and inner medullary tubules, and also increased the amount of Egr-1 positive interstitial cells. However, collagen-III expression was only repressed by furosemide treatment but did not alter by water restriction. Interestingly, however, the TGF-ß and Egr-1 producing cells were mainly localized to the outer medulla and the fornix of the papilla, rather then to the IMCD.

**Fig. 6:**
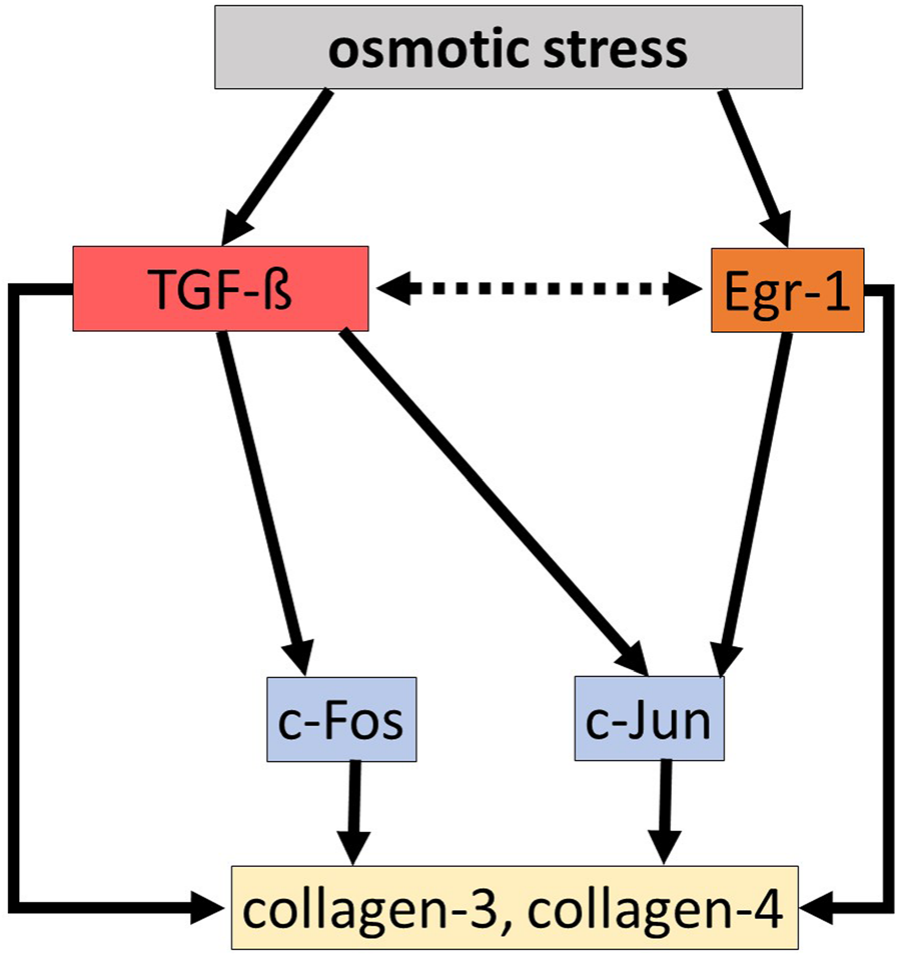
Proposed molecular effect of chronic osmotic stress on collagen production. Osmotic stress induces the expression of the profibrotic early growth response factor-1 (Egr-1), which can directly induce the transcription of collagen-III and collagen-IV. Hyperosmolarity also induces profibrotic TGF-ß expression, which can lead to AP-1 activation through cFos and cJun, therefore directly upregulate collagen expression. Egr-1 might also directly activate cJun and promote further AP-1 activation. Moreover, we postulate that TGF-ß and Egr-1 might induce each other in the renal medulla (*dotted arrows*), enhancing their profibrotic effects

In our study, chronic, sustained hyperosmolarity induced TGF-ß and Egr-1 overexpression in IMCD cells. It has been shown previously that acute hyperosmolar conditions lead to increased TGF-ß activity of rat kidney fibrobast cells in vitro, without affecting mRNA or protein expression [13]. Our slightly different results could be explained by the distinct cell type (IMCD vs fibroblast) and hyperosmolar conditions (chronic vs acute) used. TGF-ß can induce the transcription of several genes, including collagens and early response genes such as Egr-1 [30]. However, Egr-1 might also induce TGF-ß expression [14, 31] but also regulate the promoter of other genes such as both type III and type IV collagens [14, 32]. When we maintained IMCD cells in medium with normal osmolarity, TGF-ß treatment resulted in increased Egr-1 expression, as expected, and a mild increase in type IV collagen mRNA expression. As both expression levels of Egr-1 and type IV collagen were less marked in IMCD cells after TGF-ß treatment as compared to hyperosmolarity, and TGF-ß treatment failed to induce type III collagen expression, we postulate that the interplay between TGF-ß and Egr-1 overexpressions might be a key step promoting collagen synthesis in the renal medulla under hyperosmolar conditions (Fig. 6).

In our in vitro treatment protocol we wished to resemble the physiological environment of the renal medulla. The murine inner medullary collecting duct cell line (mIMCD-3) has been resported to retain most of the characteristics of IMCD cells in vivo, including the ability to adapt and grow in hypertonic medium in vitro [33]. Previous reports on IMCD cell culture in hyperosmolar or hypoosmolar environment used urea as the osmotic stressor, since urea is membrane-permeant, therefore it is not considered a hypertonic stressor as compared to NaCl [29]. We treated the cells with the combination of urea and NaCl for two reasons: first, we wished to model the in vivo milieu as IMCD cells in the renal papilla have contact with high concentration of both urea and NaCl; second, the combination of urea and NaCl significantly enhances IMCD cell survival as compared to urea alone at 900 mOsm [34]. Furthermore, our method of gradually increasing medium osmolarity for 6 days up to 900 mOsm allowed the cells to slowly accomodate to the hyperosmolar environment in contrast to previously reported acute hyperosmolar stress models, where the majority of the cells would have died within 24 h [34, 35]. Thus, we were able to study the effects of sustained hyperosmolarity, which better resembles the in vivo environment.

Taken together, sustained hyperosmolar conditions induced Egr-1, TGF-ß and AP-1 expression in the renal medullary cells both in vivo and in vitro. These profibrotic factors upregulated, in turn, the gene expression of collagens, which was abrogated by hypoosmolar conditions.

## Conclusions

To the best of our knowledge, this is the first study to show that sustained hyperosmolar conditions maintained by water restriction induced the expression of Egr-1, TGF-ß and AP-1 component cFos in the renal medulla, which might consequently upregulate the gene expression of collagens. In vitro, sustained hyperosmolarity increased the expression of Egr-1, TGF-ß and collagens in IMCD cells. This indicates that hyperosmolar milieu favors the biological processes leading to fibrosis.

As a limitation, in this study we did not test the effect of Egr-1 and/or TGF-ß inhibition during sustained osmotic stress to IMCD cells. In addition, we observed slightly different localization of Egr-1 and TGF-ß immunostaining in the renal medulla versus the IMCD cells, as a consequence of differences between our in vivo versus in vitro model system [36].

## Additional file

Additional file 1: ARRIVE Guideline Checklist. Description: ARRIVE Guidelines for reporting animal research (PDF 1078 kb)

## Abbreviations

AP-1: Activator protein-1
Col3a1: Gene of type III collagen alfa chain
Col4a1: Gene of type IV collagen alfa chain
ECM: Extracellular matrix
Egr-1: Early growth response factor-1
ESRD: End-stage renal disease
IMCD: Inner medullary collecting duct
MTT: 3-(4,5-dimethyl-2-thiazolyl)-2,5-diphenyl-2H-tetrazolium bromide
Tgfb1: Gene of transforming growth factor-ß1
TGF-ß1: Transforming growth factor-ß1
TIMP-1: Tissue inhibitor of matrix metalloprotease-1
